# Do Two Weeks in a Learning Camp after Ninth Grade Make a Difference? Experiences of Demotivated Boys with an Increased Risk of School Dropout

**DOI:** 10.3390/bs14030189

**Published:** 2024-02-28

**Authors:** Gro H. Ramsdal, Rolf Wynn

**Affiliations:** 1 Department of Social Education, Faculty of Health Sciences, UiT The Arctic University of Norway, N-9037 Tromsø, Norway; gro.h.ramsdal@uit.no; 2 Department of Clinical Medicine, Faculty of Health Sciences, UiT The Arctic University of Norway, N-9037 Tromsø, Norway; 3 Department of Education, ICT and Learning, Østfold University College, NO-1757 Halden, Norway

**Keywords:** dropout prevention, positive psychology, character strengths, visible learning

## Abstract

School dropout may have important negative consequences for the individual as well as for society. Because school grades in lower secondary education are essential for the completion of upper secondary school, remotivating demotivated ninth graders with an increased risk of dropping out seems vital. This study focuses on the experiences of Norwegian ninth grade boys at a learning camp aimed at preventing school dropout through increasing school engagement, learning, and well-being before tenth grade. We interviewed 17 of the 29 participants in one particular camp to study their experiences and analyze how they were related to the theoretical underpinning of the camp. The participants described the learning camp as a motivation boost, focusing on experiences with academic progress and increased self-regulation, factors aligning with central theoretical underpinnings of the intervention. The participants placed “connecting with others”, as in peers and teachers, among the top two factors that contributed to their re-motivation, well-being, and academic progress.

## 1. Introduction

School dropout is a significant social problem due to its deleterious consequences [1], such as unemployment, low wages, incarceration, mental health problems, drug abuse, long-term disability, and dependence on social security [2,3,4,5]. 

Statistics Norway has recently estimated the general completion rate in Norway to be 81% [6]. The present study was carried out in two rural counties north of the polar circle where the completion rates were somewhat lower, 73.6–78.1% [7], clearly below the graduation rate of 85% desired by the European Union [8]. According to DeWitte and colleagues ([8], p. 14), the numbers may be somewhat misleading due to the diversity of standards used to measure dropout and completion and the plurality of criteria underlying these measures, but they nevertheless provide the following definition of school dropout: “leaving school without obtaining a minimal credentials (most often a higher secondary education diploma)”. This dropout event is understood as the result of a gradual disengagement process [2,9]. Studies suggest that not attending school regularly, having problems with misbehavior, or minimizing the effort to learn and achieve are all indicators of an increasing disengagement from school and thus predictive of school dropout [10,11,12]. Frequent school absences or school absenteeism referring to excusable or inexcusable absences from school are found to contribute to the disengagement process by having immediate and long-term negative effects on school achievement as well as on social functioning and graduation rates [13]. A concept related to disengagement is school alienation, describing how students gradually distance themselves from school by developing negative attitudes toward learning and school activities, a concept that is also related to meanings like loss of self, apathy, loneliness, and despair [14].

Identifying the predictors of the gradual disengagement process ending in school dropout is a challenging task due to the overwhelming array of factors involved in this process, both individual factors and institutional factors. Nevertheless, there seems to be a consensus in the research community that four factors are of particular importance: school achievement, family background, school engagement, and school context [15]. First, Rumberger and Lim [2] concluded in a comprehensive review that early academic performance seemed to be among the most consistent school dropout predictors, thus making retention a central risk factor in these disengagement processes [16,17]. However, retention is almost never used in the Norwegian school system, and students stay with their original class and are provided with individually adapted teaching. Moreover, not only early achievement could predict school dropout, but also more recent negative achievement experiences can make an impact [8]. Second, there is the factor of family background, including the structure the child is surrounded by and the resources accessible but also the family practices and whether these practices support development and academic performance [2]. Third, the motivation for and active involvement in school, also called school engagement, is a strong predictor of school achievement and dropout [18,19], including cognitive, emotional, and behavioral engagement in academic, social, and extracurricular activities [20,21] but also factors like the class size, teacher–pupil ratio, teachers’ expectations, teacher support, and instructional quality [8]. Finally, there is the context of where education takes place to consider, redirecting the focus away from the individual student to the way that society organizes its system of education, from student composition, resources, and structural characteristics to processes and practices, factors that are insufficiently explored [9]. Rumberger and Lim [2] concluded that stimulating positive school climate and promoting educational policies and practices that increase school engagement is an essential factor in the prevention of school dropout.

In the search for effective practices to prevent school dropout, much attention has been paid to the role of motivation and the reasons why individuals decide to engage or disengage in school activities [22]. Eccles and Wigfield [22] call attention to expectancy–value theory, involving individuals’ beliefs about their competence, like trusting their ability to organize and effectively perform the behavior necessary to produce a particular valued outcome, also called self-efficacy. Furthermore, motivation is influenced by how individuals explain their successes and failures as being within or outside of their own control [23]. If students perceive their achievement outcomes as controlled by forces outside their influence, this might initiate or intensify disengagement processes. Theories also make a distinction between intrinsic and extrinsic motivation, underlining the intrinsic motivational energy originating when activities are perceived as interesting and enjoyable in themselves [24]. Motivation researchers have also focused on the association between children’s achievement goals and achievement behavior, finding that learning goals or mastery goals keep children more interested in difficult tasks and monitoring their own progress, while performance goals make them more interested in outperforming others [25]. 

The motivation research described above indicates that the way individuals think, feel, and behave are essential in engagement and disengagement processes, suggesting that self-regulation plays a significant role in these processes. According to Eccles and Wigfield [22], self-regulation theories underscore the significance of self-efficacy beliefs, causal attribution, and goal setting when trying to understand how task behavior is regulated. Self-regulated learners are characterized by their engagement in important processes like monitoring their own activities (self-observation), evaluating how well they perform compared to others or a standard (self-judgement), and reactions to performance outcomes, like making academic failure inspire more effort [26]. Some motivation researchers have focused on the interaction between motivation and cognition in self-regulated learning. Pintrich et al. [27] outlined a model encompassing components like student entry characteristics like the prior achievement level, social aspects like interaction between the teacher and student in class, and several motivational constructs like expectancies, values, and affect and cognitive constructs like self-regulatory and metacognitive strategies. Both types of constructs are presumed to influence engagement processes and thus academic achievement. Our interest in the prevention of school dropout is partly due to an increase in youth receiving disability benefits, particularly in the group of men below 20 years in Norway [28]. This could suggest that dropping out of school may be associated with particularly negative consequences for young men, as fewer boys (77.4%) than girls (84.2%) complete upper secondary school in Norway [6]. Researchers speculate that the greater part of this gender difference could be related to boys having lower grades than girls in primary school [29].

The transition from lower to higher secondary school seems particularly challenging to school engagement and therefore crucial to school achievement and the completion of upper secondary school. Lower school engagement and higher absenteeism in 10th grade are predictors of the non-completion of upper secondary education in Norway [30]. Students with low grades and high absenteeism are often ignored in school, possibly because they are overshadowed by students with an even higher risk of dropout, who have more serious behavioral and academic problems [1]. By identifying and supporting the students with low grades and high absenteeism before they drop out, it may be possible to help them re-engage and stay in school [1,31].

In Norway, a relatively new school dropout prevention intervention, called “Guttas Campus” (The Boy’s Camp), works to help increase school engagement of demotivated boys. This is a group intervention aspiring to give 9th grade boys with deteriorating grades and frequent absences from school and thus a high risk of dropping out of upper secondary education the academic and social support required for re-igniting their school engagement and contributing to a successful transition into and completion of upper secondary school. These learning camps are held in various parts of the country and the camp center is usually situated in a rural area within a couple of hours away from the participants’ homes. The camp management provides transportation to the camp center where the participants eat, sleep, do schoolwork, and enjoy their spare time activities together for two weeks.

The intervention consists of three elements: First, the boys participate in a learning camp based on explicit expectations, rules, and agreements for a group of ca. 25–40 participants [32,33]. The learning camp is focused on academic progress and the boys work systematically with reading, writing, and math 4–5 h a day during a 14-day learning camp. All learning activities have a specific structure and clear objectives. Because a main goal is learning to learn, all learning activities are individually customized with a teacher–student ratio of 1:5. The participants are tested before camp and thus start working in their proximal zone of development in math, reading, and writing. Teachers are focused on visible learning and all progress and completed tasks are registered on learning steps or posters hanging on the wall to be applauded by the group, giving information to the teachers and strengthening self-efficacy in the participants. The learning camp is also aimed at inspiring self-care and thus centering on healthy habits like adequate sleep, balanced diet, physical activity, socializing, and a limited use of mobile phones. The last component is a focus on values and motivation, providing a strategy for learning to learn and to cope with educational challenges, including an explicit day-to-day focus on seven character strengths, namely social intelligence, perseverance, optimism, gratitude, zest/enthusiasm, curiosity, and self-regulation [32].

Second, for one and a half years after the learning camp, the boys participate in group activities at mentor centers two times a month. The mentor centers are designed to support and motivate the participants to uphold the positive habits from the camp, help with homework, stimulate academic interest, and continue working with the meta strategies of character strengths and promote their social well-being. Third, the parents participate in mandatory parent meetings before and after the learning camp.

This model has been evaluated in Norway and Denmark. The results suggest that participants experience substantial academic improvement and lower dropout rates during lower secondary school and during the first two years of higher secondary school [32,34,35,36]. Nevertheless, there is a need for the further investigation of the outcomes of this intervention and how these outcomes relate to the theoretical underpinning of the intervention [32].

The theoretical basis of the intervention rests on the four underpinnings of positive psychology, positive education, character strengths, and visible learning [32]. Positive psychology is concerned with the relevance of positive emotional experiences and optimal functioning and declares that raising children is about more than fixing their problems. As an alternative, they encourage a focus on “identifying and nurturing children’s’ strongest qualities”, thus aspiring to develop interventions aimed at enhancing well-being ([37], p. 2). From this effort to bring positive psychology into the educational system, the movement called “positive education” emerged. 

Positive education “seeks to combine the principles of Positive Psychology with best practice teaching and with educational paradigms to promote optimal development and flourishing in the school setting“ ([38], p. 147). Seligman [39] suggested a pathway for this integration process called PERMA theory, focusing on pleasurable experiences, fostering engagement in positive activities, enhancing relationships, promoting meaning and purpose, and supporting accomplishments. To accommodate criticism concerning the risk of overemphasizing personality-based explanations and underestimating the importance of context and negative feelings, Ciarrochi and colleagues [40] proposed “context-focused positive psychology (CCP) interventions”. This model was intended to help young people face their challenges and make choices while staying true to their values, such as relating to others, challenging oneself and being curious to learn, engaging in physical activity, and caring for oneself. 

One concept from positive psychology is given particular attention in the GC intervention, namely character strengths. According to Peterson and Seligmann ([41], p. 11), “everyone brings something to the situation” and most important is character in the form of positive traits. There are six classes of such traits or virtues, namely wisdom, courage, humanity, justice, temperance, and transcendence, and they are based on 24 character strengths. The character strengths are seen as routes to displaying virtue so that courage can be achieved through character strengths like using one’s will-power to accomplish a goal or persist despite resistance or adversity. Focusing on character strengths related to various virtues, GC chose the following seven: social intelligence, perseverance, optimism, gratitude, zest/enthusiasm, curiosity, and self-regulation. These seven strengths seem to be in accordance with the competency clusters named 21st century skills by the National Research Counsil: “interpersonal competencies”, “intrapersonal competencies”, and “cognitive competencies” [42]. 

The fourth theoretical underpinning of this intervention is the concept of visible learning as described by John Hattie [43]. Reviewing the research on conditions of successful learning, Hattie found the overall message to be that teaching must be visible to the student and that the learning outcomes of such teaching must be visible to the teacher. For the teaching to be visible to the student, the teacher must become directive while cautiously listening to and watching the response of the student, thus scaffolding learning, and contributing to the development of meta-strategies for learning [43].

The camp has in various ways integrated these theoretical underpinnings in the camp activities and program. First, the camp is aimed at generating positive experiences and triggering positive emotions by providing the participants with varied activities to inspire academic and social mastery experiences, outside and inside the classroom. Second, the camp ideology draws on former experiences with the positive social and emotional outcomes of education programs such as Outward Bound [44], with central ideas including engaging in physical activity and caring for oneself. Moreover, the program is inspired by the comprehensive research on the effect of socioemotional learning and its positive effects on reading and mathematics [45]. Acknowledging the participants’ contributions to the peer group through “Man of the day” awards and using character strengths actively in the classroom, by teaching participants how to use will-power and self-regulation when struggling with math tasks, thus learning to challenge themselves, are some examples of how the theoretical underpinnings were actualized in the program. Building relationships and inspiring curiosity to learn through individualized teaching, frequent teacher support, working from the proximal developmental zone and visualizing learning through learning steps in math, progress posters in reading and writing, and cheering on each other’s visualized progress are other examples. A more detailed description of how the theoretical basis is used in the group intervention is given in a prior publication [32]. 

As noted above, there are many predictors of school dropout and completion; in this study, however, we are restricting ourselves to investigate the factors emphasized in the theoretical foundation for a learning camp called “Guttas Campus”. To better understand if and how this learning camp with its unique theoretical basis can influence the participants’ learning, school engagement, and well-being, it is adamant to investigate their experiences with this intervention. We, in this study, interviewed half of the boys participating in the 2022 “Guttas Campus” in northern Norway [34]. The aim of this study is to explore how these participants describe their experiences and how they explain the outcomes of this learning camp. Moreover, we aim to explore how the participants’ experiences are related to the theoretical underpinnings of the program and if these experiences are in accordance with the aims of the intervention.

## 2. Materials and Methods

### 2.1. Ethics and Permissions

The Norwegian Agency for Shared Services in Education and Research reviewed and approved this study (approval number 435186). Participation was voluntary and all the participants gave their written informed consent before the camp started and again in connection with the first interview. As the participants were minors, written informed consent was also sought from their parents/legal guardians.

Before we go on to describe the recruitment process, it seems relevant to identify our preconceptions related to the effects of learning camp experiences, also called ‘bracketing’ [46]. Both authors are mental health professionals with more than ten years of work experience in the field. These experiences may explain the authors’ cautious expectation concerning the potential of changing peoples’ behavior, emotions, motivations, and experiences, and their preconception that such change takes time. Thus, the authors were somewhat uncertain about which changes could be achieved in only 14 days when working with demotivated, adolescent boys. Although none of the authors have worked in learning camps, they have worked together through the last 15 years with research related to understanding the school dropout processes in adolescence and had no bindings to the learning camp.

### 2.2. Recruitment

The participants of this study were recruited from the 29 boys taking part in the “Guttas Campus” 2022 in northern Norway. These boys were 15–16 years old, completing the last months of ninth grade, and lived in small towns and rural areas attending seven different schools. The invitation to participate came mainly through their schools, and parents and teachers collaborated to suggest suitable candidates based on observations of increased absenteeism, gradually deteriorating grades, and/or social problems reducing their school engagement, thus increasing their risk of dropping out of upper secondary education [34]. Some of the boys themselves recognized their lacking school engagement and understood the positive potential of participating and therefore did not need further persuasion although they were not necessarily enthusiastic about going to camp. Other participants were persuaded by their parents and/or teachers that this learning camp could help improve their academic mastery and school engagement.

The learning camp was advertised through local newspapers, social media, and information letters to schools, politicians, and educational administrators. The camp administration held local information meetings attracting parents, potential participants, teachers, headmasters, and various counsellors, school psychologists, school nurses, etc. In these meetings, one of the authors also informed attendees about this research study. Schools were contacted by the camp administration, who informed them about the intervention and this research study, and teachers and parents were encouraged to suggest potential participants. Together with the local schools, the camp administration evaluated the applicants to find those most suited for and in need of this type of learning intervention. 

Youth who were offered a place in the camp had, over several years, experienced academic problems, eventually performing under the class average in one or several of the following competencies: math, reading, and writing. Some had social problems that hampered their school engagement and school achievement and some had both deteriorating grades and social problems. Boys who had serious behavioral problems or severe problems with drug or alcohol addiction were not offered a place in the camp as this intervention was not aimed at youth with such challenges. 

The boys who were found suitable were tested in math, reading, and writing to assess their baseline level and to individualize the teaching. When the boys and their parents attended a meeting before the boys went to a pre-camp weekend with the staff, one of the authors informed attendees again about this study and received written consent from the boys planning to participate and their parents. In total, 17 of the 29 participants gave their written consent. 

### 2.3. Qualitative Interviews

We interviewed all the boys at their local school, because they were residents within a large geographical area and interviewing them at school minimized their absence from class. We had contacted the schools beforehand to make an appointment for when to interview the boys and had been assigned a suitable room. The interviews took place 3–4 months after the camp was completed and lasted 60–90 min. We used qualitative semi-structured interviews. The questions in the interview guide were open-ended and allowed for follow-up questions and free dialogue. Questions tapped into the participants’ experiences with attending the camp, starting out with open questions like “How was your experience with the learning camp?”, “What was the best thing about participating in the learning camp?”, or “What did not work for you at camp?” and then asking about particular experiences like “How did you experience the learning outcome of the camp?” and then differentiating between experiences with math, reading, writing, and work habits and their experiences with learning about character strengths. We also asked specific questions about their experiences with the various elements of the camp like the amount of physical activity and reduced access to their mobile phones (one hour a day). Finally, we explored how being at camp had affected their thoughts about higher secondary education and what it was like coming back to school, potential changes in their school engagement, motivation and their well-being, and what kind of follow-up the school provided after camp. 

### 2.4. Qualitative Analysis

All 17 interviews were audio-recorded, transcribed, and analyzed in 2023. The interviews were analyzed using the method of a thematic analysis [47]. The process of the analysis was inspired by Malterud´s systematic text condensation method [47,48]. The condensation method is a cross-sectional analysis used to summarize and interpret qualitative data. 

The analysis consisted of four steps. In the first step, it was important to ‘listen to the voice of the informants’, which implied taking an open position to the data to ignore personal prejudices and avoid interpreting data according to preconceptions. The interviews were read repeatedly, and preliminary themes were formed, including “motivation”, “experiencing mastery”, and “self-regulation”.

The second step in systematic text condensation focused on moving from themes to codes, sorting, finding, and marking relevant text in a systematic review and organization of the data material [47]. Sections of the text were highlighted, and phrases or sentences of relevance were identified. Codes that described the content were created and relationships between different codes were identified. Codes like ‘experiencing mastery’ and ‘developing new self-efficacy’ and ‘increased school engagement’ were all elements of a re-motivation process. 

The third step involved condensing the material into code groups by focusing on words or expressions that the informants had used in the interviews. By comparing the codes, patterns were recognized, and the codes were organized into groups, thus condensing the data, and constituting the main themes of ‘Experiences of a Motivation Boost’, ‘Experiences of Academic Progress’, ‘Experiences of Connecting with Others’, and ‘Experiences of Self-Regulation’. At this stage, the main themes were compared with the interview text, to consolidate the themes and see if anything was missed. 

In step four, the analysis moved from condensation to contextualization, by drawing on concepts, theories, and prior research [47]. The emerging themes were contextualized by involving concepts from the theoretical underpinnings of the intervention [32] to help interpret the data in the context of aims and intentions and thus relating the themes to prior research. The analysis of the data was led by the first author. The second author was involved in the process through discussions regarding coding and the interpretation of excerpts. 

To enhance rigor in qualitative research and ensure that the participants’ own experiences and perspectives are presented, ‘member-checking’ is a recommended step in the analytical process [49]. This can be carried out by returning the analyzed data to the participants. However, since our participants were demotivated adolescents with reading problems, we favored another solution. When we conducted a second round of interviews (not described in the present article), we presented our analysis of the previous data, and received feedback that supported the analyses presented here.

## 3. Results

### 3.1. Introductory Remarks

Four main themes were identified: ‘Experiences of a Motivation Boost’, ‘Experiences of Academic Progress’, ‘Experiences of Connecting with Others’, and ‘Experiences with Self-Regulation’. The participants contributed many subthemes when describing their learning camp experiences. Many of the subthemes were closely related and thus contributed to the understanding of more than one of the main themes. However, the data were organized into main themes, focusing on the four subjects that the boys gave the most attention to in their interviews and to include subthemes where they seemed to have had the most impact.

### 3.2. Experiences of a Motivation Boost

Participating in a learning camp seemed to be a sensible thing to do for many of the demotivated participants, although their enthusiasm for the project varied at first. Ten out of seventeen boys said they had been persuaded by their parents to participate. While these boys understood the need to increase their presence and effort at school, they were still reluctant about coming to camp, or as one of them described it:

*I was not very interested at first, but when I saw what happened, then I thought, yes, this is good.*

Two participants described being ‘bribed’ to go to camp, because their parents were so worried about their lack of school engagement and their declining grades. However, most of the persuaded participants expressed surprise about how much they liked being at camp. One participant was promised a Playstation 5 console as a reward for completing the camp, and he described his subsequent motivation process like this:

*Then I went. And then I thought, I don’t really need Playstation 5 to participate, because this is actually really fun.*

Fourteen of the boys explicitly described a positive change in their motivation in general, for carrying out schoolwork, and for paying attention in class. One of the boys even used an English expression saying that camp was ‘a motivation boost’. This positive change in motivation had various explanations. Participants stressed the many and varied leisure time activities and being with the other boys, and some focused on the frequent breaks during school hours and a generous amount of physical activity like various ball games, canoeing, and ziplining, underscoring the importance of outdoor activities. With all this activity going on, even the school classes were described as having flow-like qualities:

*It was so much fun being there. The classes went by much faster over there than they do here (in the usual school).*

As suggested in the quotation above, the most frequently described source of a motivation boost was their feelings of mastery related to learning experiences. One of them said that the most beneficial thing at camp was

*…that I was able to learn. I had great help and they helped me understand the things I was about to learn.*

After having discovered that they were able to develop a better grasp of fractions or types of calculation or writing an essay, they talked about how they enjoyed their experiences of academic success and the fact that they had actually ‘achieved something’. One participant described his motivational boost at camp saying

*I don´t think I have ever focused so much in my entire life.*

Fourteen participants described how being able to solve the tasks presented to them made a world of difference to their motivation, and stimulated an increase in their school engagement. All of these participants explicitly described how they found it easier to be more motivated for school activities when they came back to their usual school in tenth grade. It was easier to ‘motivate yourself to do things’ after the camp. First, four participants started coming to school more and second, they focused notably more on doing the work. Seven participants talked explicitly about how they used to hate school activities like math or reading and complain about homework, and how that attitude had changed in various ways, or as one of them said about former complaining related to homework:

*I have stopped doing that, I don´t complain anymore, I just do it without my mother having to remind me.*

Coming back to school, many of them did more of the schoolwork and commented that it was really about them just having to be bothered and finding ‘the courage’ to do that. This increased engagement seemed to be related to an increased self-efficacy, a result inspired by the teachers’ optimism at camp. One of the boys said his math teacher at camp did not mind if he could not solve the math task at hand, she just tried over and over to explain until he got it. Six boys explicitly expressed that meeting such optimism made them realize that mastering school tasks was within their reach. Or as one of them expressed it:

*I just need to understand that I can do this.*

Furthermore, three participants described the discovery that when they could master the tasks, they started to like the school subjects better, for example, math. Even though some of them used to ‘hate it’, they now used words like ‘fun’ and ‘great’ and ‘loved it’. One of the boys indirectly described the motivation boost following these mastery experiences, when describing his use of his mobile phone at camp:

*I did not call my parents much…/…/but if I had learned something, then I called them.*

Eleven participants talked about the positive effects of the frequent power breaks on their school motivation and engagement, breaks that provided physical activity, and snacks in the form of fruit, vegetables, and drinks. The participants described how the power breaks made them better able to concentrate in class and helped them get rid of some of the physical unrest that many of them struggled to control in the classroom:

*I liked that we had these power breaks during classes; we had 5–10 min breaks and that helped a little. Then we could unload some energy, get some fruit and… That, I think, made me more motivated.*

One of the boys who had been persuaded to participate expressed being resentful about that and did not change his mind about camp, finding that this was not for him. He did, however, enjoy some of the leisure activities and reported that one or two things he had learned at camp might be of some use to him in his usual school. 

Summing up this main theme, we found that despite some boys being persuaded to participate, all participants but one ended up enjoying the camp, describing a motivation boost related to experiencing mastery, and developing new self-efficacy and school engagement, finding it easier to motivate themselves after the camp. The importance of frequent breaks and physical activity between classes also contributed to a motivation boost at camp.

### 3.3. Experiences of Academic Progress

When asked about their general experiences with the learning camp, fifteen of the participants spontaneously revealed that they had ‘learned a lot’, and two had learned a few useful things in math. The 15 boys who had learned a lot described differences in academic mastery before and after the camp and for some, the academic progress at camp had been substantial compared to their learning experiences at their usual school:

*As I told those who interviewed me at camp, I have learned more there than I did for two years in school.*

Furthermore, for 16 of the boys, their Experiences of Academic Progress at camp also changed the learning experiences at their usual school after camp. They described improved school achievements; some had improved their grades in a particular subject while others had increased their average grades and some reported decreased absenteeism, better concentration in class, and more completion of school tasks. 

For 15 of the participants, there was something about the way they were taught school subjects at camp that resulted in learning experiences very different from the ones they were used to in their usual schools. Or as one of them expressed it:

*It was about how they taught the subjects; they made it much easier to understand, more fun.*

One didactic strategy described as contributing to their academic progress was that learning started in their zone of proximal development. Thus, they were involved in tasks that they had the necessary skills to solve with minimal assistance. Four of the boys even used the word ‘individualization’, when praising this teaching strategy. The teachers at camp explained to the individual and not to the whole class, and one of them added, if you explain to the class then ‘only some students get it’. Another participant supported him, saying

*I got my help, not somebody else’s help.*

A second didactic strategy described as contributing to academic progress was the accessibility of teacher support at camp. At school, participants were used to a lot of waiting to receive the teacher´s attention and in the meantime, they became bored and distracted in ways, often leading to mischief. At camp, however, teachers were ready to help or as one of them stated:

*We get help when we ask for it; they check in on us all the time.*

The strategy of constantly checking in on the participants also made it unnecessary to raise one’s hand, and several boys preferred this because it did not involve revealing to the whole class that you were struggling to understand. The third didactic strategy contributing to academic progress was being allowed to work at their own pace. A fourth strategy was the ‘improved quality of teaching’. The teachers, participants recounted, explained better, and had ‘better methods’, especially in math, and were more optimistic, and this improved their learning and achievements. 

The fifth strategy contributing to academic progress, according to some of the boys, was the positive influence of ‘repetition’. They described how the learning of new skills happened too fast at their usual school. They had to move on before they mastered the skill they were supposed to learn. One participant revealed his experiences with learning math:

*At camp, I relearned almost everything. So at least now, I can remember more than I did last time.*

An important remedy in this relearning of math was the implementation of the learning step. Consequently, the sixth didactic strategy was about being able to ‘visibly follow their own academic progress’ on the wall posters, and was described as very stimulating for further effort, because they so clearly could see what they had accomplished. The visibility of progress was a strategy implemented also in reading and writing classes, where wall posters announced what they had accomplished in every single lesson. The interviews disclosed that most of the participants had read one or two books or even more in the two weeks of camp, while participating in a lot of other activities. Fourteen boys admitted that outside camp they seldom or never read books, thus revealing substantial progress in their reading activity at camp. However, despite visible feedback in the form of posters and reading tests, they did not quite seem to understand and value the significance of this progress as much as their progress in math. Other remedies were also mentioned as helpful for academic progress, like the positive influence of earmuffs on concentration in reading classes and a writing frame for structuring your essays and small tablets for doing calculations.

Finally, six participants expressed satisfaction concerning the ‘increased amount of freedom’ in learning situations and described how that contributed to academic progress. They did not have to stay at their desk in class; they could go sit with a friend and carry out math tasks together or they could relax in a sofa or a recliner when reading books and they remarked that this was relaxing and thus made reading easier and more attractive than sitting on an uncomfortable chair reading aloud to the class.

Despite the fact that their usual school environments had not changed much after the camp, it is interesting that 16 participants described their academic progress as an ongoing process 3–4 months after camp. 

Summing up, the 17 participants—with one exception—agreed that they had learned various things at camp and that there were some particular strategies used that positively influenced their academic progress in the form of the individualization of teaching, the accessibility of the teacher, and they got to work at their own pace and experienced an improved quality of teaching through better methods, learning by repetition and monitoring their own academic progress through visible learning strategies and frequent feedback, and being more relaxed in learning situations due to an increased amount of freedom or autonomy. 

### 3.4. Experiences of Connecting with Others

Asking the participants about how they liked being at camp, one of the first answers emerging from 15 of the 17 boys was that they liked the people there. First, they enjoyed being with the other boys. They came to know boys from their own school better and most of them ‘got new friends’. One of them said

*It was nice being only with the boys, and such. Everybody had the same kind of humor, sort of.*

The distraction of girls and having to look good for them was not necessary at camp and that seemed relaxing for some of the boys. They talked about the importance of ‘having the same kind of humor’ and how this made them feel more self-reliant and at ease. One participant elaborated, saying the feeling of community was important because it made them feel less alone and enabled them to share activities and problems. The 15 participants described ‘experiences of togetherness’, getting to know each other in a new way, sharing meals, learning activities, and leisure time, and exploring new places and new skills. One of the boys elaborated what togetherness at camp meant to him:

*Sitting around the table, sharing a meal, and just talking to each other.*

The many activities at camp were described as important in connecting with the other boys, there was never time to be bored, and they had somebody to hang out with in the evenings. Surprisingly, as many as 13 boys expressed that having their phone for only one hour a day was not a big problem, mainly because there were so many activities and so many peers to hang out with, although one hour *more* could have been nice. Four participants also explained how the restricted use of mobile phones at camp had made them realize the value of ‘being physically present with peers’. One of them formulated it like this:

*If you don’t use your mobile, you use the others instead, if you get my point.*

Second, the positive influence of connecting with the grownups at camp was described as essential by 15 participants and depended heavily on ‘spending time together’. Two of the boys felt they were listened to more at camp and had experienced that rules could be re-negotiated if they had strong arguments. Two participants said the grownups at camp were kinder than those at regular school, and more understanding, and one boy praised them for sharing their own life experiences and bad choices. Nevertheless, it was the teachers that received most of the attention in the interviews. Fifteen participants were enthusiastic when talking about how ‘teachers are different at camp’, they were not as strict as at school, they were less loud, and they were just firm and told them when things are not ok, but without being irritated or angry afterwards, and they thought that the teachers seemed happier and more humoristic at camp. In some way or another, all 15 boys conveyed the motivating force of the accessible and optimistic teachers. Having the same humor and being able to joke with each other was appreciated by the boys and was described as critical in connecting and “coming to know the teachers” in ways that made a difference:

*/…/because if they have the same humor and show a bit more respect for us, it is much easier for us to show them more respect.*

Connecting with teachers when the purpose was *not* to receive a reprimand seemed to be scarce but much appreciated experiences among the participants in general and was described as contributing to a more relaxed relationship that promoted learning.

Summing up, the participants described how connecting with the other boys and the teachers and leaders at camp made them more relaxed and at ease in their learning environment. They described how they obtained new friends and enjoyed having the same kind of humor as their peers, giving rise to experiences of togetherness and re-discovering the value of being physically present with peers when being without their phones for most of the day. Participants also described connecting with the adults through spending time together and coming to know the teachers, causing them to discover that teachers at camp were different, in ways that stimulated their motivation, well-being, and achievement. 

### 3.5. Experiences of Self-Regulation

The participants also spontaneously focused on the significance of their own self-regulation and found working with character strengths to be meaningful, indicating that it was smart, “making us compete to be good” as one said. Another participant described a mental image he picked up at the introduction of the camp, describing how coming to camp meant ‘finding the courage’ to jump off his cliff of protective indifference down to his actual level of skills and then build himself up from there. This image reflected the idea that succeeding at camp depended on the development of his own character, and on his ability to self-regulate.

When answering questions about their experiences with character strengths and meta-strategies for learning to learn, the participants focused on two self-regulation strategies. First, 11 boys announced that ‘perseverance or will-power’ had been essential tools in their academic progress. One maintained that it was about ‘the will to learn’. Another participant explained

*Like… if I’m going to do a task, even if is difficult, then I still have to continue. I can’t just not be bothered!*

The participants described the significance of endurance and patience when facing difficulties or failures, and these experiences kept reappearing through the interviews. At camp, there was no such thing as giving up on learning something; one of them recounted that it was a case of ‘I can’t do this yet’. They underlined the urgency of ‘not succumbing to stupid temptations’, concentrating on themselves and their own work and not getting distracted by other students. Some of the boys disclosed that believing in yourself was crucial for self-regulation or as one of them phrased it:

*If you focus on what you’re going to do, that is perseverance. And you need to think that you can do it.*

The second strategy mentioned by five participants was the character strength called self-regulation. Some of the boys reported being easily provoked and tended to end up in quarrels and fights, resulting in dozens of teachers’ notes. Furthermore, some tended to be irritable and sulky for a long time afterwards, thus pushing peers away and disturbing relationships. At camp, they worked on some simple self-regulation strategies to learn ‘to control their emotions, thoughts, and behavior’ better:

*It was this thing about character strengths; it helped me not to punch or get sulky. I learned to count to ten and that worked.*

Others worked on self-regulating their unrest and problems with concentration. These boys reported having been scolded about their agitation or hot temper or lack of concentration at school. But they had never been taught strategies for calming themselves down before they came to camp. Two participants were accustomed to receiving teachers’ notes, because they had totally given up on self-regulating their boredom in the classroom and used to fall asleep during class. Working with strategies for self-regulation in learning situations seemed to make a difference. Having been back at school for 3–4 months, instead of the dozens of teachers’ notes that they were used to, these boys had very few if any notes at all, more than halfway into the semester.

Three of the participants described how their self-regulation improvement was partly due to what we have called ‘academic socialization’ at camp. The camp staff had talked about the importance of school, and how school could help them obtain what they wanted. One participant mentioned the significance of ‘getting one’s priorities right’. Another participant talked about his new understanding that every choice you make has consequences. If he worked well at school, he would have more hours to play football in the afternoon. Supporting these views, another participant said

*There are things that the teachers said that are stuck in my head and that helps me in everyday life.*

Four boys added optimism to their list of important character strengths, underscoring the need to be motivated and gain self-efficacy.

Summing up, the participants reported on the significance of self-regulating. They described having developed an understanding that the learning camp was about more than improving grades. For them, it was also about building character and especially through executing perseverance and will-power when faced with academic or social challenges. They had acquired the will to learn, thus succumbing to fewer temptations, and to be able to control their emotions, thoughts, and behaviors better and getting their priorities right. This type of increased self-control, they described, had enabled them to make choices in everyday life that would contribute to their well-being and school engagement.

## 4. Discussion

The purpose of this study was to investigate the experiences of boys at a 14-day learning camp and explore how these experiences were related to the theoretical underpinnings of the intervention. The main theoretical inspirations came from positive psychology, and through positive education, character strengths, and visible learning.

### 4.1. Positive Psychology

Participants at the learning camp were almost unanimous in their descriptions of a motivation boost at camp. Initially, some participants were not very motivated and admitted to being persuaded into going, but even they described experiencing positive motivational changes at camp. The strength of these positive emotions was mirrored in their choice of language, using words like ‘fun’ and ‘great’ and ‘loved it’ to describe their experiences at camp. Considering that the camp experience involved longer schooldays and a rigorous program of activities and initially their motivation was not very high, these descriptions of a ‘motivation boost’ clearly suggested that the learning camp involved an enhancement of positive emotion, as is the aspiration of positive psychology [37]. Positive psychology at the individual level is about valued subjective experiences, it is a field aspiring to develop interventions aimed at enhancing well-being [50], and positive emotions have been studied as markers of well-being and happiness [51]. Accordingly, the participants described an increase in important markers of well-being through a surge of positive emotions or a motivation boost. 

The main explanations of this surge of positive emotions or a ‘motivation boost’ were, according to the participants, experiences of mastery and increased feelings of control when working on school tasks in addition to teacher optimism and support. These positive feelings made them enjoy the school subjects more and seemed to make them more perseverant when facing adversity. Even when school tasks were challenging, the participants kept coming to class more often and showed increased concentration on their schoolwork in class when back at their usual schools. This ‘motivation boost’ and increased school engagement still present 3–4 months after the camp, and seem closely related to two of the elements predicting well-being in positive psychology, namely pleasurable experiences and engagement in positive activities [39].

### 4.2. Positive Education

The pathways for integrating positive psychology in school are associated with Seligman’s PERMA theory: focusing on pleasurable experiences, fostering engagement in positive activities, enhancing relationships, promoting meaning and purpose, and supporting accomplishments [39]. As conveyed above, most participants did report pleasurable experiences and engagement in positive activities through a motivation boost at camp and even 3–4 months later, thus contributing to the fulfillment of the first two objectives of the PERMA theory. Enhancing relationships was mirrored in our data by the main theme of “connecting with others”. Being with the other boys, sharing meals, humor, and activities, and coming to know each other better was an important source of joy and generated formulations about togetherness, feelings of community, and feeling less alone. Furthermore, the boys also described the importance of connecting with the adults at camp, building relationships with them in a new way, because they were together all the time and because the adults shared their own life experiences, listened more, and shared various activities with the boys. Teachers were experienced as different at camp. Compared to experiences from their usual schools, teachers at camp were described as more accessible, as firm but less strict and loud, and as more humorous, making the participants relaxed and more ready to learn. Thus, the participants distinctly pointed to how developing positive relationships with peers and teachers was essential to their thriving at camp, just as emphasized by the enhancing relationships factor in Seligman’s [39] PERMA theory of positive education. 

Considering the fourth factor of the PERMA theory, promoting meaning and purpose, it was more difficult to find direct expressions of such experiences. Not surprisingly, few of the 14–15-year-old boys talked explicitly about experiences of purpose in their lives. Nevertheless, almost all described the learning camp experience as motivating, educational, and fun, which may be interpreted as occurrences of meaning and purpose. Some participants explicitly reported a kind of academic socialization [52] at camp, when learning about academic values and character strengths. One participant became more aware of how school could help him obtain what he wanted in life, a second learned that all choices had consequences, and a third expressed his understanding that it was important to get one’s priorities right in life. Compared to the low school engagement described by several participants before the learning camp, the frequent descriptions of a motivation boost at camp and 3–4 months later can be interpreted as an increase in their experiences of purpose and meaning.

Furthermore, one of the most frequent statements about camp was that they had “learned a lot”. The participants communicated how accessible teachers and individualized and high-quality teaching combined with repetition and increased autonomy in the classroom promoted learning at camp and involved strong mastery experiences. This also stimulated their school engagement in the form of increased school attendance, effort, and concentration and even resulted in better grades for some, when asked 3–4 months after the camp. These descriptions are closely related to the last factor in Seligman’s PERMA theory of positive education [39], namely supporting accomplishments (S). 

That said, it is important to review the critique promoted by Ciarrochi and colleagues [40] about the peril of focusing so strongly on private experiences and how people feel and think, thus coming to underrate the influence of contextual factors. Most of the boys clearly had positive experiences at camp and described how these positive experiences contrasted with their experiences with their usual school, where teachers were far less accessible, repetition was scarce, and autonomy was quite restricted in the classroom. These challenging contextual factors at their usual schools had previously given rise to experiences of demotivation and lack of purpose and after camp they were again faced with these challenging contextual factors when returning to their usual schools. Taking into consideration that the learning camp is aimed at preventing potential school dropout more than three years into the future, the duration of their increased school engagement and well-being experiences is crucial. It remains to be seen if their motivation boost and academic progress will last longer than four months after camp, considering the contextual challenges still facing them in their usual school. Although most of the participants continued to experience that the motivation boost from camp still fueled an increase in school attendance and engagement, they did indicate that this engagement lingered despite their usual school environment being less motivating. 

Looking at key features of interventions targeting cognitive or socioemotional skills and behaviors in pre-school and school children, Bailey et al. [53] point to the fact that few of such studies follow their subjects after the intervention is completed. In general, such interventions are found to produce immediate impact on child or adolescent outcomes. Nevertheless, Baily et al. [54] found, when reviewing existing follow-up studies, that fade-out of these effects is common and often co-exists with persistence. Persistence of positive effects was found to depend on types of skills targeted, institutional constraints, and opportunities in their social context and sustaining environments. The researchers state that sustaining environments require “some sort of mechanism whereby high quality postintervention contexts sustain the effects of early intervention” ([54], p. 71). 

Keeping up the effects of the surge in PERMA factors experienced at learning camp will thus most likely require support and follow-up from outside their usual schools. This is the task of the mentor centers, and much of the persistence of positive effects and potential dropout prevention therefore seems to depend on the success of these centers in prolonging the motivation boost and school engagement of the participants.

### 4.3. Character Strengths

Seligman [37,39] declared that to realize the ambitions of positive psychology and education and thus generate and maintain well-being and positive academic ambition, it is pivotal to build good character by promoting the 24 character strengths that were declared to define good character. The learning camp, however, focused on seven strengths that the leaders found to be the most helpful in promoting personal well-being and school engagement, namely social intelligence, perseverance/will-power, optimism, gratitude, enthusiasm, curiosity, and self-regulation. 

Character strength is a rather abstract concept and making demotivated mid-adolescent schoolboys grasp the meaning and relevance of these qualities seemed to be an ambitious project. Although not all the participants remembered all seven character strengths three to four months after camp, they did remember that working with these strengths felt meaningful and made a positive contribution to their social environment, “making us compete to be good” as one of them said. Interestingly, eleven of the participants found perseverance/will-power to be the most useful character strength for them personally. They underlined the importance of using their will-power to come to class, concentrate on listening to the teacher, and carry out their school tasks even when it was boring and they struggled to master the educational challenges. The participants disclosed that perseverance in class was crucial to achieve the mastery experiences that were the main source of their motivation boost. Five participants added or focused solely on self-regulation, concentrating more on the regulation of emotions and physical unrest, and keeping out of conflict. In spite of the fact that perseverance and self-regulation were presented as two different factors in the list of character strengths, the experiences described by the participants indicated that these two factors had much in common: both being about the ability to control and regulate thoughts, behavior, and feelings and this form of self-regulation was reported to be pivotal to increase their mastery experiences in learning situations at camp and at their usual school. Surprisingly, they had not been systematically trained in how to execute such control or been taught strategies for achieving such control until they came to the learning camp. 

The participants revealed that to them the road to well-being was primarily decided by their ability to persevere when facing academic challenges and boredom, to self-regulate their thoughts, feelings, and behavior in class to be able to stay focused on the learning tasks, keep out of conflict, find mastery experiences, and stay optimistic about their future.

### 4.4. Visible Learning

The overall message in visible learning is that teaching must be visible to the student and that the resulting learning must be visible to the teacher [55]. Making learning visible was one of the main strategies to promote learning at camp, using, for example, learning ladders and posters for registering academic progress and learning. Many participants explicitly described this strategy of making learning visible as useful, but almost exclusively in math. Although they spontaneously described learning important things in writing and reading, they did not mention the registration of this learning as motivating. Somehow, their experience of what they had learned in reading and writing seemed vaguer and the registration of it did not seem to inspire the same strong mastery experiences as those described using learning ladders in math. One explanation might be that academic progress in writing and reading involves slower learning processes than those related to specific math skills, thus making the day-to-day progress less visible to the participants in a two week learning camp. Nevertheless, the visible learning process at camp may still have been useful to the teachers.

Hattie [43] describes a good teacher as aware of the learning abilities and the proximal developmental zone of their students and the teacher is thus able to support the students in ways that result in engagement and learning [43]. Consequently, the visible learning process at camp may have helped the teachers monitor the proximal developmental zone of the participants in all subjects. One of the main findings in our study was that participants experienced being taught at their own level and receiving ‘my help, not somebody else’s help’ as one of them expressed it. The participants communicated that teacher accessibility and support were far more motivating and nurturing to the learning process than what they were used to at school. Thus, keeping learning processes visible may have made it easier for the teacher to provide optimal support at camp.

Summing up the discussion, as described above, the participants did confirm the importance of several theoretical underpinnings laid down by positive psychology and positive education. However, their feedback seemed to suggest that some elements of the camp design were particularly important to inspire school engagement. First, the participants underscored the relevance of accessible high-quality teaching and optimistic self-efficacy-promoting teachers in class. Secondly, the importance of positive, activity-based, and inclusive peer environments was emphasized. Finally, the participants communicated that working steadfastly on perseverance and self-regulation was a key success factor in this learning camp.

### 4.5. Limitations

Interviewing half of the participants in a learning camp might give a representative description of how most of the boys experienced the learning camp. Nevertheless, the 17 out of 29 boys that chose to participate in this study may have been those who were most satisfied with the experience, thus explaining the quite positive experiences described in our data. However, the overall evaluation report [34] from this learning camp performed by the camp administration did not display large variations in the experiences of the camp between the study participants and the rest of boys at camp. Our data seem to align with the positive results from the overall evaluation report where all boys are asked about their experiences with the learning camp and where test results in math, reading, and writing are presented. Thus, the measurements of actual academic performance agree with the boys’ interview data on learning experiences in the evaluation report, and the evaluation report data on the quantitative and qualitative effects of the camp agree with the descriptions reported in our data. The evaluation report made by the camp administration shows progress in all three competencies for the whole group, for example, going from 26% correct answers in math before the camp to 46% correct answers after the camp [34]. Several other evaluation reports show the same results [56,57,58]. However, our study had an advantage compared to the evaluation reports because the interviewing researcher was not invested in the success of the learning camp and therefore might encourage more criticism than the administrative evaluators at camp.

As always, the question of how representative the sample is deserves some attention as it speaks to the generalizability of the findings. A sample of 17 is small and cannot be used for wide generalizations. In our study, not all at-risk students in the various schools were encompassed by the camp inclusion criteria. Nevertheless, these criteria still targeted an often-ignored group of students in long-term disengagement processes causing an increased risk of dropping out of secondary education [1,4]. Furthermore, the socioeconomic representativity of the sample was unknown to us. However, the camp was without charge, thus making it available for students of all socioeconomic backgrounds. And even if all parents may not have been engaged in persuading the boys to accept the invitation to participate, teachers and counselors were active in recruiting all those fitting the inclusion criteria, thus reducing the risk that only students with the most social capital were included. 

Doing a qualitative study, our intentions were not to make wide generalization about school dropout in general or draw conclusions about cause and effect, but rather to generate an hypothesis about this comprehensive but ignored group of school dropouts with deteriorating grades and increasing absenteeism. Our study could inspire further studies on the potential effect of short-time motivation boosts on later school engagement and school completion.

## 5. Conclusions

New and efficient ways of helping demotivated secondary school students complete high school is a prioritized task in the EU2020 strategy [59]. In Norway, one of the new additions in this line of work is called ‘Guttas Campus’. We interviewed more than half of the participants in one of these learning camps to find out if their experiences with the camp after ninth grade were focused on the same factors emphasized in the theoretical underpinnings of these learning camps [32], or if the participants had other perceptions of what had contributed to their well-being and positive education.

The participants mainly described the camp as making a difference by promoting pleasurable experiences, fostering engagement in positive activities, enhancing relationships, promoting meaning and purpose, and supporting accomplishments as described in Seligman’s PERMA theory [39]. Thus, the participant descriptions were in alignment with the theoretical underpinnings of positive psychology and positive education. However, the participants described “connecting with others” as a more salient factor when explaining their experiences of motivation and academic progress, indicating that the participants experienced ‘enhancing relationships’ as a more prominent factor than the other factors in Seligman’s PERMA theory [39]. The participants also emphasized character strengths but almost exclusively focused on the strengths related to self-regulation, highlighting their ability to persevere when faced with challenges and control their feelings and behavior to avoid conflict and losing concentration in class. Participants did experience visible learning strategies as inspiring motivation, but mainly in math, although it seems possible that working with visible learning over time might show a stronger influence of this strategy in reading and writing as well.

Rumberger and Lim [2] concluded their comprehensive review on school dropout saying that policies that increase school engagement are essential in preventing school dropout. The main goal of ‘Guttas Campus’ is to increase school engagement and well-being. The participants in our study suggested that ‘enhancing relationships’ by providing active, positive peer environments, making high-quality teachers more accessible in class, and building the character strengths of will-power and self-regulation were key success factors in developing school engagement and well-being during the camp and 3-4 months later.

## Data Availability

The data on which the study is based are unavailable due to privacy restrictions.
